# Development of a psychological management intervention protocol for colorectal cancer patients: a Delphi study on benefit finding

**DOI:** 10.1371/journal.pone.0321396

**Published:** 2025-04-16

**Authors:** Linzhi Jiang, Rongrong Liu, Fan Wang, Xingqun Tan, Liyuan Sun

**Affiliations:** 1 School of Nursing, Shenzhen University Medical Schoo1, Shenzhen University, Shenzhen, Guangdong, China; 2 Nursing Department, Zunyi Medical University, Zhuhai, Guangdong, China; Lahore Medical and Dental College, PAKISTAN

## Abstract

**Objective:**

This study aimed to develop Benefit Finding intervention protocols for colorectal cancer patients on psychological management in China.

**Methods:**

We created an initial draft of such a protocol through a comprehensive literature review and group discussions. A two-round Delphi study via WeChat correspondence was conducted, with a group of 14 Chinese experts. Experts rated and scored the importance of indicators on a five-point Likert scale.

**Results:**

The response rates for two rounds both were 100%(n=14), with an authority coefficient of 0.86. The mean value assigned to the importance of the items ranged from 4.07 to 5.00, with coefficients of variation between 0.00 and 0.17; the Kendall coefficient was 0.214 (*P*<0.05). Ultimately, we established an index system comprising 8 primary items, with respective weights of 0.133, 0.131, 0.122, 0.116, 0.129, 0.122, 0.124, and 0.124. Additionally, we identified 25 secondary items and 53 tertiary items.

**Conclusion:**

This protocol demonstrates authenticity, objectivity, and broad applicability, providing a valuable reference for future psychological care and self-management strategies for colorectal cancer patients following surgery.

## Introduction

The World Health Organization predicts that by 2030, there will be approximately 2.2 million new cases of colorectal cancer (CRC) globally, with deaths reaching as high as 1.1 million. This projection indicates an expected increase in the disease burden of about 60% [1]. In recent years, the incidence of CRC in China has gradually risen. In 2022, the incidence and mortality rates of colorectal cancer in China ranked second and fourth, respectively [2], marking it as a major disease that poses a significant threat to the health of the Chinese population.

It is well established that as patients experience disease progression, prolonged treatment, and unpredictable changes in their condition, they may develop negative psychological states such as anxiety and depression [3]. These states can adversely affect their motivation for self-management. Conversely, some studies suggest that patients can also develop positive psychological and behavioral responses during their coping processes [4]. An emerging area of positive psychology that has garnered increasing attention is Benefit Finding (BF), which can mitigate some of the inherent threats posed by negative events through cognitive restructuring [5]. Additionally, BF plays a crucial role in enhancing quality of life during stressful situations [6].

In 1983, Taylor [7] put forward the Cognitive Adaptation Theory (CAT), which serves as a crucial theoretical foundation for Benefit Finding (BF). This theory encompasses three core processes: extracting positive meaning from events, striving to regain a sense of control over the event and one’s life, and reconstructing self- esteem through self-enhancement. Derived from the Cognitive Adaptation Theory, the Cognitive Behavioral Stress Management model (CBSM) has emerged as a primary intervention approach. It focuses on enhancing BF levels in patients by leveraging cognitive - behavioral skills and relaxation techniques.

Notably, although BF has demonstrated potential in various fields, research on tailored BF interventions for postoperative CRC patients in China, especially within the CBSM framework, remains scarce. In light of this research gap, this study builds upon the strengths of previous research while addressing their limitations. We have further developed and refined an intervention protocol tailored to postoperative colorectal cancer patients.

Our aim is to promote patients’ mental health and enhance their quality of life. Specifically, this study centers on BF and constructs a psychological management intervention program suitable for postoperative CRC patients within the CBSM framework. This effort is expected to provide a solid theoretical basis for improving patients’ mental well - being and emotional self - management capabilities.

## Materials and methods

### Establishment of the research group

The research team comprised 10 members, including a postgraduate supervisor, a professor of psychology, two oncology residents, one head oncology nurse, one teaching leader, two oncology nurses, and two postgraduate students collaborating on the study.

### Selecting delphi experts

There is no definite optimal number of participants for Delphi studies; however,previous recommendations suggest a minimum of 12 participants [8]. In this study, 14 healthcare professionals and educators from Shenzhen and Zhuhai were invited to participate in the Delphi Expert Consultation, in line with the study’s objective. The selection criteria for the experts were as follows:

(1) Holding an intermediate or higher professional title;

(2) Possessing a bachelor’s degree or higher;

(3) Having more than 10 years of work experience with extensive clinical expertise;

(4) Working in clinical, nursing, or psychological roles related to cancer;

(5) Demonstrating a high motivation to participate in the study and a willingness to engage in multiple rounds of expert correspondence.

### Delphi expert consultation

#### Literature review.

**Fig 1:**
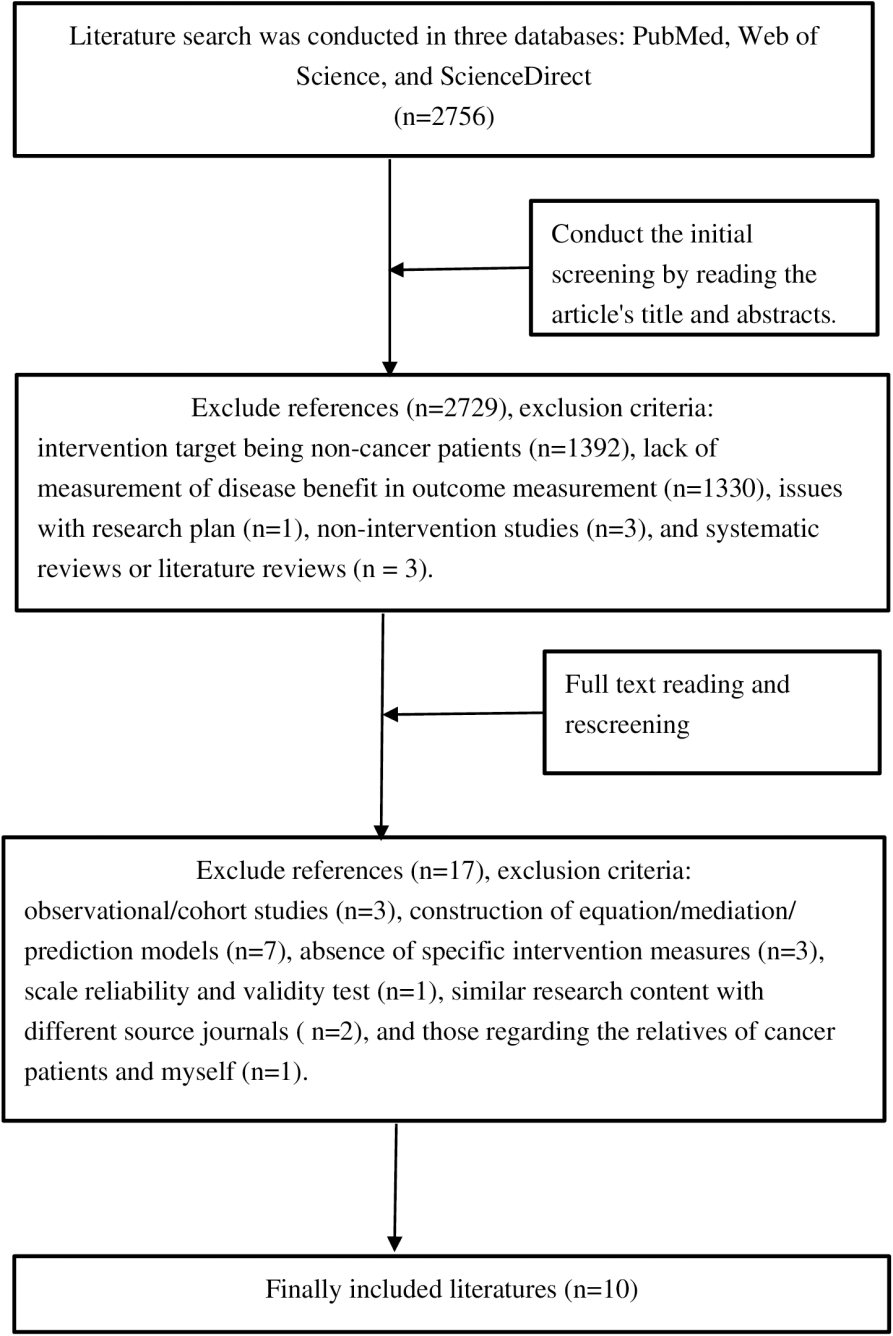
Flowchart of literature retrieval and screening.

A comprehensive search was conducted in databases such as PubMed, Web of Science, and ScienceDirect for intervention studies related to Benefit Finding (BF). The search period extended from the establishment of the database until July 2024. The search terms included: “cancer/tumor/carcinoma/neoplasm/malignancy/colorectal cancer/colon cancer/rectal cancer” “benefit finding,” and “intervene/intervention.” After systematic screening and analysis by two graduate students trained in evidence-based practice, as shown in Fig 1, a total of 10 articles were ultimately included [9–18]. The core content of the intervention protocols from the included literature was extracted and organized according to “author, intervention measures, sample sizes, measurement tools, intervention duration, evaluation time, and research conclusions,” thereby forming a draft of the intervention protocols. During literature selection and analysis, potential factors like publication bias and database limitations might affect the process. However, we’ve made every effort to ensure the literature review is comprehensive and representative.

This flowchart led to an initial search of 2756 papers through three databases, and then after two rounds of initial screening and full - text reading and rescreening, 10 papers were finally included that fit into the present study.

#### Group discussion.

The researcher convened a group meeting to present the study’s objectives, significance, technical approach, theoretical basis, and the findings derived from the literature review. Group members engaged in discussions and jointly developed the first draft of the intervention program, taking into account the presented information, their clinical experiences, and the differences in patient demographic characteristics. This draft included details such as the duration of the intervention, format, location, and personnel responsible for implementation. Once a consensus was reached, the researcher compiled and finalized the first intervention protocol based on the discussion outcomes.

#### Formation of a Delphi expert consultation questionnaire.

Following the principles of the Delphi method and the needs of this study, the consultation questionnaire was structured into three parts:

a. A basic information questionnaire for experts, including name, gender, length of service, and title.
b. An expert suggestion form regarding the intervention measures for colorectal cancer patients’ benefit finding, which included the content of the first draft of the intervention program. The importance of each entry was scored using a 5-point Likert scale, with spaces provided for modifications, deletions, or additions.
c. A questionnaire assessing the degree of authority of experts, including their familiarity with the subject matter and the basis for their judgments in completing the form.

#### Questionnaire distribution.

This study involved two rounds of Delphi expert consultation, with questionnaires distributed via WeChat to the experts. The research team summarized, analyzed, and discussed the feedback from the first round to create the second round of inquiry questionnaires. After two rounds of consultations, the experts’ opinions converged, leading to the conclusion of the inquiry process.

### Ethical consideration

The study did not need institutional review board approval as it did not affect patient care, and the information that it generated was used for developing protocols only. All participants were presented the objectives of the Delphi study, and provided their written consent by WeChat to the research group. To safeguard participant privacy, WeChat used end-to-end encryption for consent collection. Right after collection, PII (e.g., names, WeChat IDs) is removed. Participants get unique non-identifying codes, and no identifying data is used in result presentation. Team members have different access rights based on their roles.

### Statistical methods

After the questionnaires were collected, Epidata 3.1 software and SPSS 29.0 were used to establish a database employing a double-entry method. Normally distributed continuous data were expressed as means and standard deviations, while categorical data were presented as frequencies and percentages. Expert enthusiasm was represented by the response rates of the two rounds of questionnaires, and the degree of expert authority was expressed by the authority coefficient (Cr), calculated from the judgment coefficient (Ca) and the index familiarity (Cs) using the formula: Cr = (Ca + Cs)/ 2 [19].

Expert concentration was indicated by the mean of importance assignment (M), while expert coordination was expressed by the coefficient of variation (CV) and the Kendall coefficient (ω). The chi-squared test was used to verify the significance of differences, with *P*<0.05 indicating statistical significance. The weights were determined using the analytic hierarchy process (AHP) [20] to prioritize the first-level items, followed by the second-level and third-level items, with the combined weights calculated as the average of the AHP and the priority diagram method.

## Results

### Basic information of the experts

In this study, a total of 14 experts completed two rounds of inquiries, representing various institutions, including tertiary cancer hospitals, tertiary general hospitals, and higher education institutions. The working experience of the consulted experts ranged from 11 to 40 years (mean ± SD: 21.86 ± 8.82). Of the experts, 8 (57.1%) held a master’s degree or higher, and 9 (64.3%) had a deputy senior title or above, as shown in Table 1.

**Table 1:** Basic information of experts.

Variables	N (%)
**Sex**	
Male	6 (42.9)
Female	8 (57.1)
**Work years**	
10-19	7 (50.0)
20-29	4 (28.6)
30 and above	3 (21.4)
**Level of education**	
Undergraduate	6 (42.9)
Master	5 (35.7)
Doctor	3 (21.4)
**Graduate supervisor**	
Yes	2 (14.3)
No	12 (85.7)
**Title**	
Intermediate	5 (35.7)
Deputy senior	7 (50.0)
Senior	2 (14.3)
**Specialty**	
Clinical medicine	5 (35.7)
Nursing management	6 (42.9)
Nursing education	2 (14.3)
Psychology education	1 (7.1)

### Expert positive coefficient

The response rates for two rounds both were 100%, indicating a high level of enthusiasm among the experts.

### Expert authority coefficient

The judgment coefficient (Ca) and the index familiarity (Cs) were 0.90 and 0.82, respectively, resulting in an authority coefficient (Cr) of 0.86. An authority coefficient above 0.7 is generally considered to indicate a high level of authority [21]. Therefore, the experts in this study demonstrated a high degree of authority and credibility.

### Expert concentration

In the two rounds of expert consultations, the mean value assigned to the importance of the items ranged from 3.29 to 5.00 and 4.07 to 5.00, respectively. A mean value above 3.50 is typically acceptable [19], indicating that expert opinions and suggestions were adjusted after the consultations, resulting in a higher degree of consensus.

### Expert coordination coefficient

It is generally accepted that the coefficient of variation (CV) should be below 0.25 [19], with values of the Kendall coefficient (ω) closer to 1 indicating a higher the degree of coordination among expert opinions. In the first round, the CV values were ranged from 0.00 to 0.29 and the ω was 0.169 (*P*<0.001), respectively. In the second round, these values ranged from 0.00 to 0.17, with a Kendall coefficient of 0.214 (*P*<0.001), as shown in Table 2, indicating that expert opinions were becoming more consistent. Moreover, the CV in the second round was lower than that in the first round, with a statistically significant difference (*t*＝7.997, *P<*0.05), suggesting a higher level of approval for *t*he protocol among the experts.

**Table 2:** Coordination coefficient (ω) of expert opinions and test results.

Index	First round			Second round		
**ω**	**χ²**	***P***	**ω**	**χ²**	***P***
**Primary item**	0.148	14.471	0.043	0.250	24.462	0.001
**Secondary item**	0.195	49.216	<0.001	0.273	91.763	<0.001
**Tertiary item**	0.158	104.116	<0.001	0.170	124.098	<0.001
**Overall items**	0.169	175.454	<0.001	0.214	254.631	<0.001

### The analytic hierarchy process

Based on the results of two rounds of Delphi expert consultation, eight primary items were identified: “Confidence-building”, “Identification of pressures”, “Reconstructing perceptions”, “Social support”, “Emotional needs”, “ Emotional management”, “ Value of life”, and “Summary and evaluation.” We constructed an eight-order judgment matrix and conducted analyses using the Analytic Hierarchy Process. The weighted values of the eight primary items were 0.133, 0.131, 0.122,0.116, 0.129, 0.122, 0.124, and 0.124, respectively. The results are summarized in Table 3.

**Table 3:** Expert consultation results on psychological management and behavioral intervention plans for colorectal cancer patients.

Items	Importance score (mean±standard deviation)	Coefficient of variation	Weight	Combination weight
**1. Confidence-building (face to face)**	5.00±0.00	0.00	0.133	—
**1.1 Relationship maintenance during hospitalization**	4.86±0.36	0.07	0.081	0.114
**1.2 Relevance of this study**	4.71±0.47	0.10	0.079	0.096
**1.3 Leave contact details**	4.50±0.52	0.12	0.075	0.058
**1.4 Meditative Breathing Training**	4.43±0.51	0.12	0.074	0.046
**1.1.1 To introduce yourself, understand the patient’s general situation, and establish a good nurse-patient relationship**	4.64±0.50	0.11	0.077	0.080
**1.2.1 Introduce the purpose, content, modalities of the study, and obtain informed consent**	4.79±0.43	0.09	0.080	0.108
**1.3.1 Inform the patient of the next appointment for treatment or follow-up**	4.50±0.52	0.12	0.075	0.058
**1.3.2 Follow up via WeChat**	4.36±0.63	0.15	0.073	0.039
**1.3.3 Confirm patient and/or family contact details**	4.50±0.65	0.14	0.075	0.058
**1.4.1 Instruct patients to practice meditative breathing exercises and reflect on the value of life**	4.71±0.47	0.10	0.079	0.096
**1.4.2 Encourage patients to find a comfortable position to relax, and then relax their entire body**	4.57±0.51	0.11	0.076	0.071
**1.4.3 Guide patients to allow their breath to remain natural and calm**	4.71±0.47	0.10	0.079	0.096
**1.4.4 Supervise the patient’s practice at least twice a week for 15–30 minutes each session, and document their progress**	4.64±0.50	0.11	0.077	0.080
**2. Identification of pressures (face to face)**	4.93±0.27	0.05	0.131	—
**2.1 Discuss stressors**	4.36±0.63	0.15	0.070	0.055
**2.2 Provide knowledge related to stress**	4.36±0.63	0.15	0.070	0.055
**2.3 Progressive muscle relaxation training**	4.07±0.27	0.07	0.065	0.035
**2.1.1 Guide the patient to discuss current or potential problems and stressors**	4.36±0.63	0.15	0.070	0.055
**2.2.1 Present information in a lecture format for easier identification of stressors**	4.86±0.36	0.07	0.078	0.108
**2.2.2 Classify types of pressure**	4.79±0.43	0.09	0.076	0.099
**2.2.3 Identify stressors and their levels**	4.79±0.43	0.09	0.076	0.099
**2.2.4 Introduce the cognitive behavioral stress management model**	4.57±0.51	0.11	0.073	0.090
**2.3.1 Explain the progressive muscle relaxation through a video demonstration, followed by practice with the patient (15–30 minutes)**	4.43±0.51	0.12	0.071	0.071
**2.3.2 Instruct to take three deep breaths, exhaling slowly**	4.50±0.52	0.12	0.072	0.082
**2.3.3 Guide the patient to tense and relax different muscle groups sequentially**	4.36±0.63	0.15	0.070	0.055
**2.3.4 Hold tension for 7–10 seconds, then release for 15–20 seconds**	4.43±0.51	0.12	0.071	0.071
**2.3.5 Encourage mindful awareness of any residual tension in the body**	4.29±0.73	0.17	0.068	0.042
**2.3.6 Ensure patient practices at least twice a week**	4.50±0.65	0.14	0.072	0.082
**3. Reconstructing perceptions (online)**	4.57±0.51	0.11	0.122	—
**3.1 Identify misperceptions**	4.79±0.43	0.09	0.108	0.139
**3.2 Cognitive Transformation**	4.36±0.63	0.15	0.098	0.104
**3.3 Transfer relevant knowledge**	4.86±0.36	0.07	0.110	0.150
**3.4 Progressive muscle relaxation training**	4.07±0.27	0.07	0.092	0.051
**3.1.1 Facilitate discussion on patients’ understanding of the disease, rehabilitation, and treatment to identify misperceptions**	4.29±0.61	0.14	0.097	0.083
**3.2.1 Collect focused misperceptions from patients**	4.71±0.47	0.10	0.106	0.128
**3.2.2 Conduct online lectures to provide accurate information about the disease.**	4.50±0.52	0.12	0.102	0.116
**3.2.3 Foster confidence through encouragement and support to create a positive atmosphere**	4.29±0.61	0.14	0.097	0.083
**3.3.1 Encourage patients with effective disease management to assist their peers in stress management, promoting mutual support**	4.29±0.73	0.17	0.097	0.083
**3.4.1 Implement progressive muscle relaxation as outlined in sections 2.3.1-2.3.6**	4.14±0.36	0.09	0.094	0.062
**4. Social support (online)**	4.36±0.74	0.17	0.116	—
**4.1 Provide knowledge on support system**	4.36±0.63	0.15	0.088	0.056
**4.2 Example analysis method**	4.86±0.36	0.07	0.098	0.131
**4.3 Progressive muscle relaxation training**	4.57±0.51	0.11	0.092	0.108
**4.1.1 Explore changes in patients’ interpersonal relationships before and after diagnosis**	4.57±0.51	0.11	0.092	0.108
**4.1.2 Highlight the importance of interpersonal communication**	4.86±0.36	0.07	0.098	0.131
**4.1.3 Discuss methods for building networks**	4.43±0.51	0.12	0.089	0.082
**4.1.4 Provide strategies for fostering harmonious interpersonal relationships**	4.57±0.51	0.11	0.092	0.108
**4.1.5 Assess patient’s spousal relationships, family support, and financial resources**	4.36±0.63	0.15	0.088	0.056
**4.1.6 Emphasize a patient-driven approach, with study members providing assistance**	4.43±0.51	0.12	0.089	0.082
**4.2.1 Use example analyses to encourage patient with cancer to share their support experience**	4.43±0.65	0.15	0.089	0.082
**4.3.1 Implement progressive muscle relaxation as outlined in sections 2.3.1-2.3.6**	4.36±0.63	0.15	0.088	0.056
**5. Emotional needs (online)**	4.86±0.36	0.07	0.129	—
**5.1 Facilitate emotional communication**	4.86±0.36	0.07	0.138	0.186
**5.2 Guide meditative breathing exercises**	4.21±0.70	0.17	0.120	0.075
**5.3 Promote gratitude**	4.29±0.73	0.17	0.122	0.100
**5.1.1 Discuss the emotional aspects of coping with treatment**	4.50±0.52	0.12	0.128	0.165
**5.1.2 Invite patients and their families to express their thoughts and feelings together**	4.43±0.51	0.12	0.126	0.149
**5.1.3 Encourage reconnecting with family or friends to discuss emotions**	4.36±0.63	0.15	0.124	0.124
**5.2.1 Implement meditative breathing exercises as outlined in sections 1.4.1-1.4.4**	4.36±0.63	0.15	0.124	0.124
**5.3.1 Instruct patients to write thank-you letters to individuals who have helped them, fostering a sense of gratitude**	4.21±0.70	0.17	0.120	0.075
**6. Emotional management (online)**	4.57±0.51	0.11	0.122	—
**6.1 Encourage emotional journaling**	4.36±0.75	0.17	0.108	0.091
**6.2 Provide positive guidance**	4.71±0.47	0.10	0.117	0.145
**6.3 Guide meditative breathing exercises**	4.71±0.47	0.10	0.117	0.145
**6.1.1 Instruct patients to maintain an emotional journal using the ABC format (a. Stimulus; b. Thoughts and Interpretations; c. Emotional Reactions) weekly.**	4.43±0.76	0.17	0.110	0.117
**6.1.2 Suggest recording daily mood changes and their causes**	4.21±0.70	0.17	0.104	0.058
**6.1.3 Encourage sharing daily emotions with a close family member or friend and document patient reflections**	4.86±0.36	0.07	0.120	0.165
**6.2.1 Instruct patients on managing negative emotions such as anger, depression, and anxiety, while providing positive reinforcement**	4.29±0.73	0.17	0.106	0.072
**6.2.2 Encourage creating a positive artifact (e.g, handbill) based on personal interests, documenting the process to emphasize positive emotions**	4.36±0.63	0.15	0.108	0.091
**6.3.1 Implement meditative breathing exercises as outlined in sections 1.4.1-1.4.4**	4.43±0.51	0.12	0.110	0.117
**7. Value of life (face to face)**	4.64±0.50	0.11	0.124	—
**7.1 Facilitate goal-setting in life**	4.79±0.43	0.09	0.211	0.285
**7.2 Progressive muscle relaxation training**	4.29±0.61	0.14	0.189	0.114
**7.1.1 Encourage patients to share their thoughts on the meaning of life**	4.64±0.50	0.11	0.204	0.222
**7.1.2 Assist in setting short- and long-term goals based on their current situation to enhance aspirations and expectations**	4.64±0.50	0.11	0.204	0.222
**7.2.1 Implement progressive muscle relaxation as outlined in sections 2.3.1-2.3.6**	4.36±0.50	0.11	0.192	0.156
**8. Summary and evaluation (face to face)**	4.64±0.50	0.11	0.124	—
**8.1 Review and Summarize**	4.64±0.50	0.11	0.124	0.101
**8.2 Collect comments and suggestions**	4.64±0.50	0.11	0.124	0.101
**8.3 Guide meditative breathing training**	4.50±0.52	0.12	0.120	0.068
**8.1.1 Review the first seven psychological interventions**	4.64±0.63	0.14	0.124	0.101
**8.1.2 Summarize the effectiveness of positive psychological interventions based on the cognitive behavioral stress management model**	4.71±0.47	0.10	0.126	0.149
**8.1.3 Assess perceived benefits related to the disease, quality of life, and self-management**	4.71±0.47	0.10	0.126	0.149
**8.2.1 Collect patient feedback on the intervention model**	4.86±0.36	0.07	0.130	0.182
**8.3.1 Implement meditative breathing exercises as outlined in sections 1.4.1-1.4.4**	4.71±0.47	0.10	0.126	0.149

### Index revision

During the two rounds of consultations, some experts provided suggestions and comments on the items. The main changes were as follows:

(1) Deletion of items: in the first round of correspondence, four tertiary items with coefficients of variation above 0.25 were deleted.
(2) Addition of items: a total of seven items were added, including the second-level items “meditation and breathing training” and “progressive muscle relaxation training,” which were incorporated into each topic.
(3) Modification of entries: two primary items, six secondary items, and twelve tertiary items were modified. Additionally, four tertiary items were combined into two, and the analysis method for understanding the patient’s situation was adjusted accordingly for items 4.1.1 and 4.2.1.

In the second round of consultation, the mean value for all items exceeded 3.5, and the coefficient of variation for all items was below 0.25. Textual changes were made to six items for clarity, indicating that expert opinions and suggestions converged and that the consultation could be concluded. Therefore, the final intervention protocol consisted of 8 primary items, 25 secondary items, and 53 tertiary items. The details are provided in Table 3.

## Discussion

### A uthenticity and objectivity of the intervention protocol

In this study, we reviewed a substantial body of international literature on interventions related to BF in tumor patients. This review encompassed various aspects, including intervention methods, sample sizes, measurement tools, intervention durations, evaluation time, and research conclusions, allowing us to formulate a preliminary intervention framework. The research team, which possesses extensive clinical experience and theoretical knowledge, developed the initial draft of the intervention through collaborative brainstorming sessions. Experts from academia and healthcare institutions provided constructive feedback on this proposal, which was subsequently refined based on data analysis from the consultation forms. This iterative process resulted in a final draft that is both representative and reliable.

The effective response rate for the expert consultation questionnaires exceeded 90% in both rounds, and the authority coefficient was 0.86, indicating a high level of enthusiasm and authority among the experts [21]. During the consultation process, ten experts offered valuable insights and suggestions, further enhancing the credibility of the consultation results. Following the second round of consultation, the mean values for all items were above 3.50, with 31 exploratory items also exceeding this threshold. The coefficients of variation for the two rounds were 0.00–0.29 and 0.00–0.17, respectively, while the Kendall coefficients were 0.169 and 0.214 (*P*<0.001). These results indicate that expert opinions became increasingly consistent, supporting the credibility of the inquiry findings [19].

Thus, this study constructed the intervention plan through a combination of literature review, group discussions, and the Delphi expert consultation method. The favorable objective numerical values reflecting the authority and coordination of expert opinions enhance the scientific rigor and objectivity of the modified proposal, thereby establishing a solid theoretical foundation for subsequent implementation.

### The general applicability of the intervention protocol

Cognitive Behavioral Stress Management (CBSM) is a psychological treatment approach designed to help patients recognize and manage stress, ultimately improving their mental health and quality of life [22]. This method integrates various interventions, including expressive writing [10] and mindfulness [11], with a focus on stress, cognition, and emotion. It also incorporates breathing relaxation techniques and homework assignments to enhance BF levels. A literature review [9–14] has identified CBSM as one of the most widely used interventions for promoting BF, primarily through group psychological interventions. Walsh et al [11] selected 256 male patients with prostate cancer who were randomly assigned to either a 10-week CBSM intervention or a one-day psychoeducation, with BF and perceived stress management skills as the intervention targets. The results showed that CBSM significantly improved the psychosocial adaptation and immune status of the patients, effectively enhancing their stress management capabilities.

In this study, the intervention protocol is grounded in practical considerations to address patients’ emotional expression and psychological needs. It employs a combination of online and offline group interventions, including lectures [11], teaching sessions [12], and diary writing [10]. The team has developed a public account for effective health education, offering online guidance for patients and their families through classes, example analysis method, testimonials, emotional journals, and crafting activities. The offline format includes face-to-face presentations of the study, lectures, goal-setting sessions, and summary reviews. Recognizing that the target population is predominantly elderly, all formats are designed to be straightforward and easily comprehensible, with breathing relaxation techniques that are readily adopted by patients.

This protocol aims to cultivate positive emotions, shift negative cognition, and enhance quality of life through both one-to-many and many-to-many approaches, fulfilling physiological needs while also addressing the need for love and belonging. Additionally, the intervention is tailored to align with patients’ treatment plans and home schedules, making it suitable for most cancer patients. Therefore, the intervention formats for each theme are accessible and facilitate implementation, providing targeted support for patients’ mental health and self-management, particularly for those recovering from surgery.

## Limitations

This study has some limitations. After two rounds of expert consultations, the Kendall coefficients was 0.214. Although this value was higher in the second round compared to the first, the degree of coordination among expert opinions remained relatively low. Additionally, many patients undergoing surgery for colorectal cancer receive chemotherapy, which often has a prolonged duration. This situation makes intergroup contamination a potential issue, complicating the implementation of standardized, randomized controlled trials. Furthermore, some studies have indicated that while CBSM interventions demonstrate significant short-term effects, their long-term efficacy may be suboptimal [12]. Therefore, in future clinical practice, we aim to continuously optimize and improve the intervention protocol by focusing on minimizing inter-group contamination, extending the duration of the intervention, and increasing follow-up periods.

## Conclusion

This study developed an intervention protocol for the psychological management of postoperative colorectal cancer patients, based on a comprehensive literature review, group discussions, and Delphi expert consultations. This protocol provides a reference for future oncological psychological care. The research team plans to conduct quasi-experimental studies in the next phase to evaluate the effectiveness of the protocol and to further refine its components.

## References

[1] Arnold M, Sierra MS, Laversanne M, Soerjomataram I, Jemal A, Bray F. Global patterns and trends in colorectal cancer incidence and mortality. Gut. 2017;66(4):683–91. doi: 10.1136/gutjnl-2015-310912 26818619

[2] Han B, Zheng R, Zeng H, Wang S, Sun K, Chen R, et al. Cancer incidence and mortality in China, 2022. J Natl Cancer Cent. 2024;4(1):47–53. doi: 10.1016/j.jncc.2024.01.006 39036382 PMC11256708

[3] Renna ME, Shrout MR, Madison AA, Alfano CM, Povoski SP, Lipari AM, et al. Depression and anxiety in colorectal cancer patients: Ties to pain, fatigue, and inflammation. Psychooncology. 2022;31(9):1536–44. doi: 10.1002/pon.5986 35751505 PMC10278052

[4] Conley CC, Andersen BL. Lemons to lemonade: Effects of a biobehavioral intervention for cancer patients on later life changes. Health Psychology. 2019;38(3):206–16. doi: 10.1037/hea0000717 30762400 PMC6464376

[5] Kritikos TK, Stiles-Shields C, Shapiro JB, Holmbeck GN. Benefit-finding among young adults with spina bifida. J Health Psychol. 2022;27(5):1176–86. doi: 10.1177/1359105321990804 33541148 PMC10403805

[6] Lin Y, Luo X, Li J, Xu Y, Li Q. The dyadic relationship of benefit finding and its impact on quality of life in colorectal cancer survivor and spousal caregiver couples. Support Care Cancer. 2021;29(3):1477–86. doi: 10.1007/s00520-020-05602-x 32699998

[7] Taylor SE. Adjustment to threatening events: A theory of cognitive adaptation. American Psychologist. 1983;38(11):1161–73. doi: 10.1037/0003-066x.38.11.1161

[8] Milner SH, Feltbower RG, Absolom KL, Glaser AW. Identifying social outcomes of importance for childhood cancer survivors: an e-Delphi study. J Patient Rep Outcomes. 2024;8(1):14. doi: 10.1186/s41687-023-00676-7 38315438 PMC10844160

[9] Antoni MH, Lehman JM, Kilbourn KM, Boyers AE, Culver JL, Alferi SM, et al. Cognitive-behavioral stress management intervention decreases the prevalence of depression and enhances benefit finding among women under treatment for early-stage breast cancer. Health Psychol. 2001;20(1):20–32. doi: 10.1037//0278-6133.20.1.20 11199062

[10] Rosenberg AR, Bradford MC, Barton KS, Etsekson N, McCauley E, Curtis JR, et al. Hope and benefit finding: Results from the PRISM randomized controlled trial. Pediatr Blood Cancer. 2019;66(1):e27485. doi: 10.1002/pbc.27485 30270489 PMC6249081

[11] Walsh EA, Antoni MH, Popok PJ, Moreno PI, Penedo FJ. Effects of a randomized-controlled trial of cognitive behavioral stress management: Psychosocial adaptation and immune status in men with early-stage prostate cancer. Gen Hosp Psychiatry. 2022;79:128–34. doi: 10.1016/j.genhosppsych.2022.10.012 36375341 PMC9729459

[12] Groarke A, Curtis R, Kerin M. Cognitive-behavioural stress management enhances adjustment in women with breast cancer. Br J Health Psychol. 2013;18(3):623–41. doi: 10.1111/bjhp.12009 23210527

[13] McGregor BA, Antoni MH, Boyers A, Alferi SM, Blomberg BB, Carver CS. Cognitive-behavioral stress management increases benefit finding and immune function among women with early-stage breast cancer. J Psychosom Res. 2004;56(1):1–8. doi: 10.1016/S0022-3999(03)00036-9 14987957

[14] Penedo FJ, Molton I, Dahn JR, Shen B-J, Kinsinger D, Traeger L, et al. A randomized clinical trial of group-based cognitive-behavioral stress management in localized prostate cancer: development of stress management skills improves quality of life and benefit finding. Ann Behav Med. 2006;31(3):261–70. doi: 10.1207/s15324796abm3103_8 16700640

[15] Ratcliff CG, Milbury K, Chandwani KD, Chaoul A, Perkins G, Nagarathna R, et al. Examining Mediators and Moderators of Yoga for Women With Breast Cancer Undergoing Radiotherapy. Integr Cancer Ther. 2016;15(3):250–62. doi: 10.1177/1534735415624141 26867802 PMC4972683

[16] Chaves C, Vázquez C, Hervás G. Positive interventions in seriously-ill children: Effects on well-being after granting a wish. J Health Psychol. 2016;21(9):1870–83. doi: 10.1177/1359105314567768 25637070

[17] Zhang M-M, Chen J-J, Zhang T, Wang Q-L, Li H-P. Feasibility and effect of a guided self-disclosure intervention designed to facilitate benefit finding in breast cancer patients: A pilot study. Eur J Oncol Nurs. 2021;50:101879. doi: 10.1016/j.ejon.2020.101879 33338740

[18] Mo Q, Tan C, Wang X, Soondrum T, Zhang J. Optimism and symptoms of anxiety and depression among Chinese women with breast cancer: the serial mediating effect of perceived social support and benefit finding. BMC Psychiatry. 2022;22(1):635. doi: 10.1186/s12888-022-04261-y 36199048 PMC9533572

[19] Ge J, Zhao C, Lu J, Zhang X, Zhou X, Wang R, et al. A Delphi Study to Construct an Index of Practice for Community Nurses Providing Transitional Home Care for Patients with Chronic Diseases. Inquiry. 2024;61:469580241246474. doi: 10.1177/00469580241246474 38666736 PMC11089844

[20] Zhang C, Ma E-L, Liu B-L, Wu B, Gu Z-C, Lin H-W. Framework Development for Clinical Comprehensive Evaluation of Drugs-a Study Protocol Using the Delphi Method and Analytic Hierarchy Process. Front Pharmacol. 2022;13:869319. doi: 10.3389/fphar.2022.869319 35662698 PMC9161709

[21] Qu H, Shewchuk RM, Chen Y-Y, Richards JS. Evaluating the quality of acute rehabilitation care for patients with spinal cord injury: an extended Donabedian model. Qual Manag Health Care. 2010;19(1):47–61. doi: 10.1097/QMH.0b013e3181ccbc2a 20042933

[22] Wang F, Zhang S, Song B, Han Y. Anxiety, depression, and quality of life in postoperative non-small cell lung cancer patients under the intervention of cognitive-behavioral stress management. Front Psychol. 2023;14:1138070. doi: 10.3389/fpsyg.2023.1138070 37325749 PMC10264623

